# Increasing Monounsaturated Fatty Acid Contents in Hexaploid *Camelina sativa* Seed Oil by *FAD2* Gene Knockout Using CRISPR-Cas9

**DOI:** 10.3389/fpls.2021.702930

**Published:** 2021-06-29

**Authors:** Kyeong-Ryeol Lee, Inhwa Jeon, Hami Yu, Sang-Gyu Kim, Hyun-Sung Kim, Sung-Ju Ahn, Juho Lee, Seon-Kyeong Lee, Hyun Uk Kim

**Affiliations:** ^1^Department of Agricultural Biotechnology, National Institute of Agricultural Sciences, Rural Development Administration, Jeonju-si, South Korea; ^2^Department of Biological Sciences, Korea Advanced Institute of Science and Technology, Deajeon, South Korea; ^3^Department of Bioenergy Science and Technology, Chonnam National University, Gwangju, South Korea; ^4^Department of Bioindustry and Bioresource Engineering, Plant Engineering Research Institute, Sejong University, Seoul, South Korea

**Keywords:** monounsaturated fatty acids, FAD2, CRISPR-Cas9, genome editing, camelina

## Abstract

Seed oils are used as edible oils and increasingly also for industrial applications. Although high-oleic seed oil is preferred for industrial use, most seed oil is high in polyunsaturated fatty acids (PUFAs) and low in monounsaturated fatty acids (MUFAs) such as oleic acid. Oil from Camelina, an emerging oilseed crop with a high seed oil content and resistance to environmental stress, contains 60% PUFAs and 30% MUFAs. Hexaploid Camelina carries three homoeologs of *FAD2*, encoding fatty acid desaturase 2 (FAD2), which is responsible for the synthesis of linoleic acid from oleic acid. In this study, to increase the MUFA contents of Camelina seed oil, we generated *CsFAD2* knockout plants via CRISPR-Cas9-mediated gene editing using the pRedU6fad2EcCas9 vector containing *DsRed* as a selection marker, the U6 promoter to drive a single guide RNA (sgRNA) covering the common region of the three *CsFAD2* homoeologs, and an egg-cell-specific promoter to drive *Cas9* expression. We analyzed *CsFAD2* homoeolog-specific sequences by PCR using genomic DNA from transformed Camelina leaves. Knockout of all three pairs of *FAD2* homoeologs led to a stunted bushy phenotype, but greatly enhanced MUFA levels (by 80%) in seeds. However, transformants with two pairs of *CsFAD2* homoeologs knocked out but the other pair wild-type heterozygous showed normal growth and a seed MUFAs production increased up to 60%. These results provide a basis for the metabolic engineering of genes that affect growth in polyploid crops through genome editing.

## Introduction

Plants contain a large proportion of polyunsaturated fatty acids (PUFAs), such as linoleic acid and α-linolenic acid, in their membrane lipids and storage lipids. Seed oils are primarily used as edible oils, but their industrial use has been gradually increasing (Biermann et al., 2011). Seed oils have excellent potential for industrial applications because their major constituents are C16-C18 fatty acids, which are similar in chemical structure to diesel oil (Durrett et al., 2008). Monounsaturated fatty acids (MUFAs) such as oleic acid are preferred for industrial uses, for example as biodiesel fuels and biolubricants, due to their higher thermal-oxidative stability and viscosity relative to other common fatty acids (Smith et al., 2007; Davis et al., 2008). Besides industrial uses, high oleic seed oils are favorable for deep frying (Warner and Knowlton, 1997). Therefore, high-oleic vegetable oil is preferred for both industrial and food uses. However, most vegetable oils contain high PUFA and low MUFA levels.

Many studies have been conducted to artificially alter the fatty acid composition of oilseed crops based on their intended uses. Given that fatty acid desaturase 2 (FAD2) is responsible for the synthesis of linoleic acid from oleic acid (Okuley et al., 1994), high-oleic oilseed crops have been developed by suppressing *FAD2* gene expression using ethyl methane sulfonate-induced mutagenesis (Lee et al., 2017), antisense RNA (Sivaraman et al., 2004; Jung et al., 2011), RNA interference (RNA*i*) (Nguyen et al., 2013; Chen et al., 2015; Wood et al., 2018), and combined antisense and RNA*i* technologies (Nguyen and Shanklin, 2009; Lee et al., 2016). Genome editing techniques such as the clustered regularly interspaced short palindromic repeats (CRISPR)-CRISPR-associated protein (Cas) system (Ran et al., 2013) have recently been used to more effectively perform knockout (KO) of *FAD2* to increase MUFA contents (Braatz et al., 2017; Jiang et al., 2017; Morineau et al., 2017; Okuzaki et al., 2018; Do et al., 2019; Huang et al., 2020). Genome editing generated a double strand break (DSB) using site-specific nucleases such as zinc-finger nucleases (ZFNs), transcription activator-like effector nucleases (TALENs), or Cas at specific locations in genomic DNA, which is subsequently repaired via non-homologous end joining (NHEJ) or homology-directed repair (HDR) (Mahfouz et al., 2014).

Camelina (*Camelina sativa*) is an emerging oilseed crop with several outstanding agronomic traits, including a short generation time (within 100 days), strong resistance to drought and low temperature stress, and low requirements for water and fertilizer (Bansal and Durrett, 2016), which make it suitable for cultivation on marginal lands. Camelina seeds contain high levels of oil (30–49%) with a fatty acid composition of ~50–60% PUFAs and 30% MUFAs (Vollmann and Eynck, 2015). Camelina oil is mainly used as a biofuel (such as biodiesel and jet fuel) and as a raw material in the chemical industry (Berti et al., 2016). In addition, due to its easy transformation via the floral dip method (Lu and Kang, 2008; Liu et al., 2012), Camelina is considered an ideal platform for the production of industrial lipids and for plant lipid biotechnology research (Kagale et al., 2014; Bansal and Durrett, 2016; Malik et al., 2018).

Camelina is an allohexaploid plant (2*n* = 6*x* = 40) belonging to the mustard family (Kagale et al., 2014) that carries three *FAD2* homoeologs sharing over 97% homology (Hutcheon et al., 2010; Kang et al., 2011). The Camelina genome consists of two highly similar subgenomes with seven chromosomes and a third subgenome with six chromosomes that is slightly less similar to the other two (Hutcheon et al., 2010; Kagale et al., 2014). Although editing the allohexaploid genome of Camelina requires considerable time and effort, genome editing of hexaploid wheat has already been performed successfully (Wang et al., 2014).

In this study, we generated triple *CsFAD2* KO Camelina mutants by CRISPR-Cas9-mediated gene editing using one single guide RNA (sgRNA) specific to the three *CsFAD2* homoeologs. These lines showed a dramatic increase in seed MUFA levels of ~80%, but they had a stunted bushy phenotype and produced much fewer seeds than the wild type. However, Camelina plants with one intact *CsFAD2* homoeolog grew normally and yielded seed oil with a 60% MUFA content. Together, these results suggest that Camelina requires one functional *CsFAD2* homoeolog for normal growth and that the number of functional *CsFAD2* homoeologs affects MUFA content in seed oil in a dose-dependent manner.

## Materials and Methods

### Plant Materials and Growth Conditions

*Camelina sativa* cv. CAME and cv. Suneson were used as wild-type parents for *CsFAD2* KO using 35S promoter-driven *Cas9* (35SP:*Cas9*) and EC1.2 promoter-driven *Cas9* (EC1.2P:*Cas9*), respectively. The plants were grown in a culture room at 19°C under long-day conditions (16 h light/8 h dark).

### sgRNA Design

**Figure 1:**
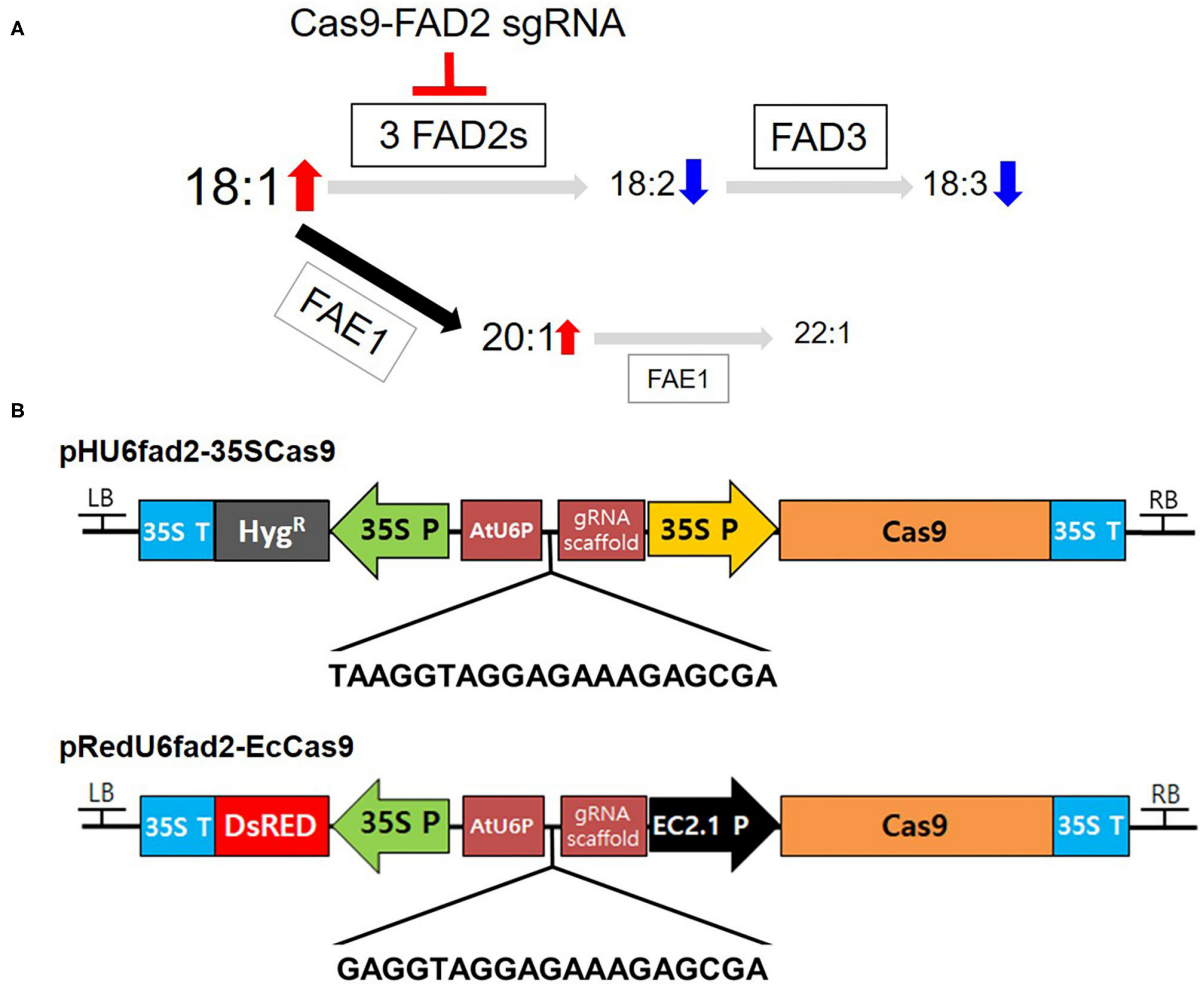
Binary vectors for *CsFAD2* KO in Camelina. **(A)** Simplified schematic diagram of genes encoding enzymes involved in fatty acid synthesis and metabolism in Camelina seeds. Cas9-FAD2 sgRNA was constructed to edit three copies of *FAD2* genes in Camelina. Seed fatty acids are synthesized sequentially from 18:1 to 18:2 by FAD2 and 18:3 by FAD3. In addition, 18:1 is synthesized as 20:1 and 22:1 by FAE1. The font size of fatty acids represents the increase and decrease in fatty acid content predicted as a result of *FAD2* editing. Gray arrows indicate a decrease and black arrow indicates an increase in metabolic flow. **(B)** The pHU6fad2-35SCas9 construct containing the *hygromycin phosphotransferase* (*Hyg*^R^) gene as a selection marker and the 35S promoter (35S P) to drive *Cas9*. The pRedU6fad2-EcCas9 construct containing *DsRed* as a selection marker and the egg-specific EC1.2 promoter to drive *Cas9* expression. Both vectors contain the AtU6 promoter (AtUP6) and a gRNA scaffold for sgRNA expression. The nucleotide sequence between AtU6P and the gRNA scaffold is a sequence of sgRNA for priming all *CsFAD2* genes.

The region of the coding sequence of the *CsFAD2-1* homoeolog (GenBank accession No. HQ008320) from bp 1 to bp 300 was used as the target sequence. The criteria for sgRNA selection using Cas Designer (Park et al., 2015) (http://rgenome.net/cas-designer) were as follows. First, GC content range was from 40 to 60%. Second, out-of-frame score was 67 point or higher. Third, sgRNA candidates with 1- to 3-base mismatches were rejected to prevent off-target effects. Fourth, three on-targets had to be present in that enabled the KO of all three pairs of *CsFAD2* homoeologs using one sgRNA. Based on the selected sgRNA, the primers used for sgRNA insertion were designed differently and the sequences are listed in Supplementary Table 1. The sequences of sgRNAs for the vector described in Figure 1B.

### Vector Construction

The sgRNA was inserted into the vectors as described above (Kim et al., 2016). Ten μL of a pair of primers for 100 μM sgRNA were mixed and annealed in a PCR machine under the following conditions. 95°C for 5 min, linear gradient from 95 to 25°C for 70 min, and hold at 10°C. The vectors were digested with *Aar*I (New England Biolabs, MA, USA) between the AtU6 promoter and the guide RNA scaffold, and the annealed sgRNA primer pair and the digested vector were ligated together using T4 DNA ligase (New England Biolabs). The ligated clones were transformed into *E. coli* DH5α. The primer sequence used for sequencing of the inserted sgRNA is listed in Supplementary Table 1.

To generate a vector containing *DsRed, phosphinothricin acetyltransferase* gene as known as *BAR* (Basta resistance) gene in the pMDC123 vector (Curtis and Grossniklaus, 2003) was removed by digestion with *Xho*I. Given that the *DsRed* gene contains an *Xho*I recognition sequence, the *DsRed* gene was amplified using a primer containing a recognition site of *Sal*I compatible with *Xho*I and digested with *Sal*I. The two digested fragments were ligated together using T4 DNA ligase. The sequences of the primers used this procedure are listed in Supplementary Table 1. The *Cas9* gene in pHAtC was amplified and inserted into the pDONR211 vector using Gateway BP Clonase^TM^ (Invitrogen, CA, USA). The *Cas9* gene in the entry clone was transferred into the pMDC123 vector in which the *BAR* gene was replaced with *DsRed* by LR reaction using LR Clonase^TM^ II (Invitrogen, CA, USA).

The egg-cell-specific promoter was amplified based on the nucleotide sequence of EC1.2 (At2g21740) (Wang et al., 2015) using *Arabidopsis* genomic DNA as a template and forward and reverse primers that included *Eco*RI and *Spe*I recognition sequences, respectively. Both PCR products and the vector were digested with *Eco*RI and *Spe*I, and ligated. The sequences of the primers used to clone the EC1.2 promoter are listed in Supplementary Table 1. Following this procedure, the vector was digested with *Eco*RI and the fragment containing the *AtU6* promoter and the gRNA scaffold was inserted into this vector. SgRNA was inserted into the complete binary vector harboring the EC1.2 promoter and *Cas9* using the method described above.

### Camelina Transformation

*Agrobacterium tumefaciens* (GV3101 strain) cells were transfected with the desired construct using the freeze-thaw method (Hofgen and Willmitzer, 1988). Unlike the vacuum infiltration method for Camelina transformation described previously (Lu and Kang, 2008), a simple floral dip method based on the method used for *Arabidopsis* transformation with slight modifications was employed (Liu et al., 2012).

In brief, when the Camelina flowers opened, Agrobacterium harboring the vector described above was inoculated in 1 L of LB broth containing spectinomycin (50 μg/mL) and rifampicin (50 μg/mL) and incubated with shaking (200 rpm) for 24 h. The cells were harvested when OD_600_ = 0.8 and resuspended in transformation solution [5% (w/v) sucrose, 0.05% (v/v) Silwet L-77]. The floral organs of Camelina were soaked in transformation solution containing Agrobacterium cells for 30 s, and the whole plant was covered with a black plastic bag for 16–24 h. Three rounds of floral dipping were performed at 1-week intervals.

### Selection of Transgenic Plants/Seeds

Transformed seeds with the hygromycin resistance gene (encoding hygromycin phosphotransferase) were surface-sterilized, sown on Murashige and Skoog (MS) medium containing 30 mg/L hygromycin, 1% (w/v) sucrose, and 0.8% (w/v) plant agar, and incubated at 25°C under long-day conditions (16 h light/8 h dark) for 2 weeks. The surviving plants were transferred to soil. Transformed seeds with the *DsRed* gene were screened based on orange fluorescence using a green light and a red filter in the dark (Lu and Kang, 2008).

### Targeted Deep Sequencing

Primers were designed so that the predicted DSB point would be included in the center and the melting temperature (T_m_) value of the *CsFAD2* homoeolog-specific sequence would be 55°C. The amplicon size (except for non-specific *CsFAD2* homoeologs) was 293 bp, and the primer sequences are listed in Supplementary Table 1. The composition of the first PCR mixture for amplification of the target gene was as follows. Template genomic DNA (5 ng/μL) 2.5 μL, 1 μM primer forward and reverse 5 μL each, 2X KAPA HiFi Hotstart readymix PCR kit (Roche, Swiss) 12.5 μL, total volume of 25 μL. The conditions for the first PCR were 95°C for 3 min; 25 cycles of 95°C for 30 s, 55°C for 30 s and 72°C for 30 s; and 72°C for 5 min. PCR purification was performed when a sample of the PCR products was electrophoresed in a 1% (w/v) agarose gel and only the desired size band was visible. Nested PCR was performed using an ILMN NexteraXT index kit v2 (FC-131-1002; Illumina, CA, USA). The composition of the second PCR mixture was as follows. Purified first PCR products 2.5 μL, 1 μM index primer forward and reverse 2.5 μL each, 2X KAPA HiFi Hotstart readymix PCR kit 12.5 μL, water 5 μL, total volume of 25 μL. The second PCR conditions were 95°C for 3 min; 8 cycles of 95°C for 30 s, 55°C for 30 s and 72°C for 30 s; and 72°C for 5 min. As with the first PCR, the PCR product was purified when only a band of the desired size appeared on upon electrophoresis in a 1% agarose gel. The PCR product was purified and adjusted to a concentration of 1 ng/μL. Samples of purified PCR product >5 μL were used for targeted deep sequencing with the MiSeq system (Illumina, CA, USA). The resulting fastq files were analyzed for nucleotide sequences and mutations using Cas analyzer (http://rgenome.net/cas-analyzer) (Park et al., 2017).

### Homoeolog-Specific PCR Using Genomic DNA

Genomic DNA was extracted from up to 96 Camelina samples at the same time using a magnetic bead type Genomic DNA Prep Kit for Plants (BIOFACT, Republic of Korea) following the manufacturer's instruction. In brief, <100 mg of tissue was sampled from Camelina leaves at three different sites and placed in a 2 mL microcentrifuge tube containing 2.3 mm chrome beads (BioSpec Products, OK, USA). The leaf samples were frozen in liquid nitrogen and ground with a Tissue Lyser (Qiagen, Germany). Lysis buffer was added to the 2 mL microcentrifuge tube. Following centrifugation, the supernatant was transferred to a 96 deep well-plate. Isopropanol and magnetic beads were combined and transferred to a 96 deep well-plate. Magnetic beads and attached nucleic acids, proteins, and so on were separated with a 96 multi magnet pipet and washed three times in a 96 well-round bottom plate, and the genomic DNA was eluted. Using this DNA as a template, three different PCRs were performed for each individual with homoeolog-specific primers. After confirming the PCR products by electrophoresis, the PCR products were purified with a magnetic bead type PCR Purification Kit (BIOFACT, Republic of Korea) for Sanger sequencing following the manufacturer's instructions. The sequences of homoeolog-specific primers are listed in Supplementary Table 1. The sizes of the *CsFAD2* homoeolog-specific amplicons were sequentially 465 bp, 415 bp, and 417 bp.

### Analysis of Indel Ratios Based on Sanger Sequencing Results

The ab1 files generated from Sanger sequencing of wild type (WT) and genome-edited Camelina were analyzed with the web-based tool TIDE (Brinkman et al., 2014) (http://tide.deskgen.com) or DsDecodeM (Liu et al., 2015) (http://skl.scau.edu.cn/dsdecode/).

### Fatty Acid Analysis

The fatty acid composition and oil content of seeds were determined by gas chromatograph (GC). Approximately 10 mg of seeds were weighed and crushed in a 2 mL microcentrifuge tube containing 2.3 mm chrome beads and 0.5 mL toluene using a Tissue Lyser (Qiagen, USA). The material was transferred to a glass tube with a polytetrafluoroethylene-sealed cap and combined with 0.5 mL of 5% (v/v) H_2_SO_4_ in methanol containing pentadecanoic acid (15:0) as an internal standard. Following fatty acid extraction and transmethylation at 85°C for 90 min, 1 mL of 0.9% (w/v) NaCl was added to the sample, and fatty acid methyl esters (FAMEs) were extracted three times with 0.5 mL of *n*-hexane. The FAMEs were dried with nitrogen gas, dissolved in 0.2 mL *n*-hexane, and analyzed by GC-2010 plus (Shimadzu, Japan) GC with a flame ionization detector (FID) and a 30 m × 0.25 mm (inner diameter) HP-FFAP column (Agilent, USA) while the oven temperature was increased from 150 to 220°C at 30°C/min and then held for 11 min. Highly pure nitrogen gas was used as the carrier gas.

### Measurement of Seed Size and 100-Seed Weight

The picture containing seeds and ruler was taken by optical microscope and the seed size in the picture was measured using ImageJ software. One-hundred seed weight was determined in triplicate. *CsFAD2* triple mutant EC#5-6-14-3 had 29 seeds, therefore its 100-seed weight was calculated by 29-seed weight^*^100/29.

### Transmembrane Domain Prediction

The transmembrane domains of the *CsFAD2* homoeologs were predicted using the TMHMM server (http://www.cbs.dtu.dk/services/TMHMM/) and TOPCONS (http://topcons.cbr.su.se/).

## Results and Discussion

### Construction of Two Vectors Containing Single Guide RNAs for *CsFAD2* KO

To increase the levels of oleic acid, a representative MUFA, produced in the fatty acid metabolism in Camelina seeds, we constructed two versions of Cas9-FAD2 to simultaneously edit all three pairs of *CsFAD2* homoeologs (Figure 1A). Using Cas Designer (Park et al., 2015), we selected an sgRNA sequence for KO of all *CsFAD2* homoeologs using the criteria described in the Materials and Methods. The sequence of the sgRNA is 5′-TAAGGTAGGAGAAAGAGCGA(GGG)-3′ [where the parenthetical sequence at the 3′ end is the protospacer adjacent motif (PAM)], which is located at the +150 position and in the—direction. We inserted this sgRNA into the pHAtC vector (Kim et al., 2016), which contains a hygromycin resistance gene, the AtU6 promoter (driving sgRNA expression), and human codon-optimized *SpCas9* from *Streptococcus pyogenes* under the control of the cauliflower mosaic virus 35S promoter. The complete vector was named pHU6fad2-35SCas9 (Figure 1B).

The 35S promoter, which drives *Cas9* expression in the pHAtC vector, is expressed strongly in vegetative tissues such as leaves but weakly in reproductive organs. It may therefore be relatively ineffective for genome editing compared to an egg-cell-specific promoter such as the EC1.2 promoter (Wang et al., 2015; Zhang et al., 2016). To select Camelina plants with antibiotic or herbicide resistance genes requires a large amount of plant culture medium, large space in the culture room, and considerable labor. In contrast, seeds containing the *DsRed* gene can be easily selected on the day of harvest (Lu and Kang, 2008), and thus growing plants from these seeds requires much less time, space, and labor than growing and selecting herbicide-resistant plants. Therefore, we constructed a vector harboring the egg-cell-specific EC1.2 promoter and the *DsRed* gene as a selection marker (see Materials and Methods). New sgRNA sequence was slightly modified from old one. From 20 nt of old sgRNA, 2 nt of 5′ end were removed because there was no significant difference of editing efficacy between 20 nt- and 17 nt-target binding sequences (Lv et al., 2019) and add G at 5′ end for reducing off-target effect (Fu et al., 2014; Moon et al., 2019). Sequence of new sgRNA was 5′-GAGGTAGGAGAAAGAGCGA(GGG)-3′ (parenthetical sequence is PAM). In this vector, one more G was added to the 5' end of the sgRNA rather than pHU6fad2-35SCas9 to reduce the off-target effect without reducing the on-target activity (Cho et al., 2014). The complete vector harboring the sgRNA was named pRedU6fad2-EcCas9 (Figure 1B).

The sgRNA sequences used in two previous studies involving Camelina *CsFAD2* KO with the CRISPR-Cas system were different from those used in the current study (Jiang et al., 2017; Morineau et al., 2017). In one study, oleic acid content was successfully increased in genome-edited Camelina using any of three sgRNAs: CsFAD2 R1, CsFAD2 R2, or CsFAD2 F1 (Jiang et al., 2017). In the other study, when two sgRNAs (sgRNA1 and sgRNA2) were used, sgRNA2 induced mutations more efficiently than sgRNA1 (Morineau et al., 2017). Although the mutation-inducing effects of these sgRNAs might have differed due to the differences in their sequences, when all three sgRNAs in the first study and sgRNA2 in the second study were expressed under the control of the AtU6 promoter, *CsFAD2* gene editing was successful. Meanwhile, in the second study, sgRNA1 under the control of the AtU3 promoter was less successful in inducing mutations of *CsFAD2* homoeologs in Camelina. Therefore, it appears that the AtU6 promoter leads to stronger sgRNA expression than the AtU3 promoter (Zhang et al., 2016; Morineau et al., 2017).

### EC1.2 Promoter-Driven Cas9 Is More Efficient at Inducing Indel Mutations Than 35S Promoter-Driven Cas9 in Camelina

We transformed flowering Camelina using Agrobacterium harboring one of the binary vectors by the floral dip method. Seeds transformed with pHU6fad2-35*SC*as9 (SC) were surface-sterilized and sown on MS medium containing hygromycin. Six surviving individuals, which we named SC#1 to SC#6, were transferred to soil and cultured. We isolated genomic DNA from the leaves of these plants and performed targeted deep sequencing. Six plants, SC#1 to SC#6, showed an indel ratio <1%, whereas SC#1 had a relatively high indel ratio of 16.2%. SC#1 also had a 13% ratio of +T mutations at the DSB point (5′-CCC_PAM_TCG/CTC-3′) in the *CsFAD2-1* homoeolog, suggesting that *CsFAD2-1* is a heteroallelic mutant.

**Figure 2:**
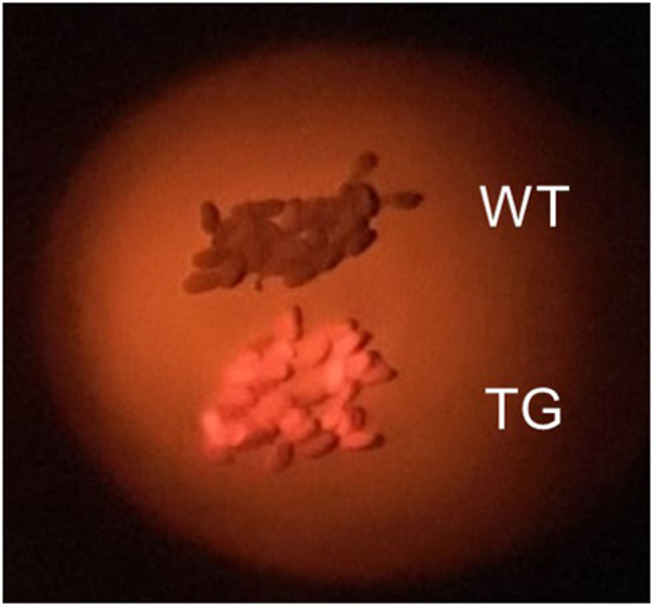
Selection of transgenic Camelina seeds. Wild-type (WT) Camelina seeds appear brown and transgenic (TG) seeds containing DsRed appear orange-red when photographed using a green LED and a red filter in the dark.

We immediately observed seeds transformed with pRedU6fad2-*E*c*C*as9 (EC) to detect DsRed fluorescence, selected 65 T_1_ seeds (EC#1 to EC#65) with orange-red fluorescence (Figure 2), and sowed them in soil. Sanger sequencing following genomic PCR of T_1_ plants revealed that the indel ratio ranged from 0.4 to 84.1% (Supplementary Table 2). The average indel ratio was 17.5% and three individuals with <1% of indels that were considered as WT. In addition, 17 transgenic plants had an indel ratio of 30% or more, accounting for ~26% of all individuals. Overall, insertions were 3-times more common than deletions, and 1 nt insertions were observed at the highest rate. However, high ratios of indels without frameshift mutations were also observed in many individuals.

For 35SP:*Cas9*, indels occurred in only one out of six individuals, whereas for EC1.2P:*Cas9*, indels occurred in 93.8% of individuals. In addition, for 35SP:*Cas9*, the highest indel ratio we observed was 16.2%, whereas for EC1.2P:*Cas9*, the highest indel ratio was 84.1%. Therefore, in germ cells, the expression of *Cas9* under the control of the EC1.2 promoter, rather than the 35S promoter, is much more efficient at inducing DSBs, resulting in more efficient mutagenesis. These results are in line with the observation that homoallelic mutants were efficiently created in *Arabidopsis* within one or two generations when the expression of *Cas9* was driven by the EC1.2 promoter (Wang et al., 2015).

### *Cas9* Expression Driven by the 35S Promoter Fails to Induce Mutations Effectively in Camelina

We measured the indel ratios in the T_2_ progeny of SC#1. The indel ratios of 7 of the 10 individuals were <2%, and thus they showed little difference from WT. Of the three remaining individuals, the indel ratios of SC#1-4 (19.2%), SC#1-5 (55.4%), and SC#1-9 (40.3%) were higher than that of SC#1 (16.2%). SC#1-4, SC#1-5, and SC#1-9 all contained +T in the *CsFAD2-1* homoeolog at different ratios (43.6, 95.2, and 94.1%, respectively), suggesting that SC#1-4 is a heteroallelic mutant and SC#1-5 and SC#1-9 are homoallelic mutants of the *CsFAD2-1* homoeolog. SC#1-5 also contained 48.4% of –CTC at the 3′ position of the DSB in the *CsFAD2-2* homoeolog, which was not the case for SC#1. This finding suggests that the CTC deletion was newly generated in SC#1-4.

To investigate how the higher indel ratios in T_2_ plants compared to their T_1_ ancestors affect the fatty acid composition of seed oil, we performed fatty acid analyses. Compared to WT, the T_3_ seeds from the three T_2_ plants (SC#1-4, SC#1-5, and SC#1-9) showed slight changes in MUFA and PUFA contents, but they were all within the margin of error (Supplementary Table 3). Therefore, maximum 55.4% *CsFAD2* KO (SC#1-5) was insufficient to cause changes in fatty acid composition in Camelina seeds. That is, the presence of only three active *CsFAD2* homoeologs was sufficient to maintain a fatty acid composition like that of WT Camelina, which has three active *CsFAD2* homoeolog pairs. We generated T_3_ offspring from the three T_2_ individuals and subjected them to Sanger sequencing (Supplementary Table 4). Forty progeny of SC#1-4 contained +T in the *CsFAD2-1* homoeolog, and these lines showed negligible levels of deletions. The highest indel ratio was 43.5%, and +T was detected in approximately below 7% (7 progenies), 17–23% (26 progenies), and 35–39% (13 progenies) of all descendants of SC#1-4. These results suggest that the +T mutation in *CsFAD2-1* was inherited in WT, heterozygous, and homozygous form similar to the 1:2:1 genotypic ratio predicted for Mendelian segregation and that significant additional indels no longer occurred. Most descendants of SC#1-5 had +T mutations at a ratio of 30.7–36.5% and –CTC at a ratio of 17.7–42.5%, and eleven individuals had only +T mutations at a ratio of 33.7–36.1%. These results suggest that the +T mutation in the *CsFAD2-1* homoeolog was homozygous and that the –CTC mutation in *CsFAD2-2* was heterozygous or homozygous. The maximum indel ratio observed was 89.7%, and eight individuals had an indel ratio of 80% or more. All descendants of SC#1-9 had the +T mutation at a ratio of 32.2–37.6%, but only one had a low proportion (3.0%) of deletions. These results also suggest that the +T mutation in the *CsFAD2-1* homoeolog was homozygous. Therefore, all three lines inherited their indel types from the parental generation through Mendelian inheritance. The 48 offspring of SC#1-5 contained +T *CsFAD2* homoeologs at a ratio of 33%, suggesting that a certain *CsFAD2* homoeolog pair located in one subgenome was mutated with a T insertion. At the same time, we detected 11 offspring without –CTC, 30 individuals with ~17% –CTC, and 8 individuals with ~40% –CTC. These results indicate that the WT:heterozygote:homozygote ratio for the *CsFAD2-2* homoeolog in SC#1-5 was 11:30:8. These ratios are also similar to the 1:2:1 genotypic ratio predicted for Mendelian segregation like the case of *CsFAD2-1* homoeolog in SC#1-4. The genotypic ratio was confirmed to be 1:2:1 by the χ^2^ test (*P* > 0.05). The T_2_ generation of SC#1 had an indel ratio of 16%. The increase in the indel ratio in the two T_2_ progeny indicated that mutations in the intact *CsFAD2* homoeologs continued to occur, as a result of further Cas9 activity, but at low frequency, and the genotype was primarily determined by the parental genotype. This finding is in line with previous reports (Feng et al., 2014; Wang et al., 2014; Mao et al., 2016). It is likely that the egg-cell-specific promoter causes mutations more efficiently than the 35S promoter, as mentioned above (Wang et al., 2015).

Since the 3 nt deletion did not cause a frameshift, and resulted in only an amino acid deletion, the generation of complete *CsFAD2* KO Camelina plants with increased MUFA contents could not be ensured. Thus, no further experiments were performed with the SC Camelina lines.

### *CsFAD2* KO Results in Increased MUFA Contents in Camelina Seeds

We harvested and planted seed from EC#5, which showed the highest indel ratio (Supplementary Table 2). To analyze indels in each homoeolog in hexaploid Camelina more precisely, we designed *CsFAD2* homoeolog-specific primers (see Materials and Methods) using the region of this gene showing differences in the 5′ untranslated region and the intron sequence upstream of the start codon, because the coding regions of the *CsFAD2* homoeologs share almost the same sequence (Kang et al., 2011). Using these primers, we performed *CsFAD2* homoeolog-specific genomic PCR and Sanger sequencing of WT Camelina, which confirmed that the PCR products were specific to each homoeolog. Therefore, we analyzed the nucleotide sequence and indel ratio for each homoeolog in EC#5 and its descendants showing a high indel ratio and estimated their genotypes (Table 1). EC#5 had a 1 nt insertion present at a ratio of 48.8%, a 5 nt deletion at a ratio of 18.9%, and a 3 nt deletion at a ratio of 15.5% in all *CsFAD2* homoeologs (Supplementary Table 2). Following reanalysis by homoeolog-specific PCR, we estimated that *CsFAD2-1* was a biallelic mutant with a +A and –CTC mutation, *CsFAD2-2* was a biallelic mutant with a +T and –GCTCT mutation, and *CsFAD2-3* was a heteroallelic mutant with a +T mutation. The descendants of EC#5 had the following characteristics. For *CsFAD2-1*, there were a biallelic mutant and a homoallelic mutant of +A or –CTC. For *CsFAD2-2*, similar to *CsFAD2-1*, there were also biallelic mutants and homoallelic mutants of +T or –GCTCT. For *CsFAD2-3*, there were five WT, six +T heteroallelic mutants, and two +T homoallelic mutants, as well as one chimera. Therefore, the EC#5 genotype was inherited in a Mendelian manner.

**Table 1:** Genotypes of Camelina progenies of EC#5 using Sanger sequencing following homoeolog-specific PCR.

**Line**	**FAD2-1**		**FAD2-2**		**FAD2-3**		**Genotype**
	**Zygosity**	**Mutation**	**Zygosity**	**Mutation**	**Zygosity**	**Mutation**	
EC#5	bi.	1i+3d	bi.	1i+5d	het.	1i	aa′bbCc
EC#5-30	hom.	1i+1i	hom.	5d+5d	chimera	chi(d8, d6)	aabbCC
EC#5-42	hom.	1i+1i	hom.	5d+5d			aabbCC
EC#5-14, #5-28	bi.	1i+3d	bi.	1i+5d			aa′bbCC
EC#5-3, #5-5, #5-45	bi.	1i+3d	hom.	5d+5d			aa′bbCC
EC#5-46	hom.	3d+3d	bi.	1i+5d			a′a′bbCC
EC#5-19, #5-23	hom.	3d+3d	hom.	5d+5d			a′a′bbCC
EC#5-1, #5-20	hom.	1i+1i	bi.	1i+5d	het.	1i	aabbCc
EC#5-4, #5-33, #5-38, #5-47	bi.	1i+3d	bi.	1i+5d	het.	1i	aa′bbCc
EC#5-6, #5-35	bi.	1i+3d	hom.	5d+5d	het.	1i	aa′bbCc
EC#5-7, #5-13, #5-26	bi.	1i+3d	hom.	1i+1i	het.	1i	aa′bbCc
EC#5-8	hom.	3d+3d	bi.	1i+5d	het.	1i	a′a′bbCc
EC#5-44	hom.	3d+3d	bi.	1i+5d	het.	1i	a′a′bbCc
EC#5-12	bi.	1i+3d	hom.	1i+1i	hom.	1i+1i	aa′bbcc
EC#5-29	bi.	1i+3d	bi.	1i+5d	hom.	1i+1i	aa′bbcc

*1i, 3d, and 5d indicate 1 nt insertion, 3 nt deletion, and 5 nt deletion, respectively. Bi., chi, het., and hom. represent biallelic, chimeric, heteroallelic, and homoallelic mutants, respectively. Uppercase letters, lowercase letters, and lowercase letters with ′ in the Genotype column represent an intact CsFAD2 gene, a nonsense CsFAD2 mutant gene, and a missense CsFAD2 mutant gene with a 3 nt deletion, respectively. A, B, and C in the Genotype column represent CsFAD2-1, CsFAD2-2, and CsFAD2-3, respectively*.

The genotypes of both EC#5-12 and EC#5-29 for the homoeologs *CsFAD2-1*/*CsFAD2-2*/*CsFAD2-3* were +T, –CTC/+A, –GCTCT/+T, +T; thus, they were triple mutants of the three *CsFAD2* homoeologs. For plants with the –CTC mutation, which causes a missense mutation, we expected that *CsFAD2* KO would have little effect. However, both EC#5-12 and EC#5-29 showed a stunted bushy phenotype and died before seeds could be harvested, and thus showed same phenotype as the recently reported *CsFAD2* triple mutants (Morineau et al., 2017). Therefore, it is likely that the single amino acid deleted due to –CTC had an important impact on the activity of CsFAD2. For a detailed discussion, see sixth subsection of Results and Discussion section.

**Figure 3:**
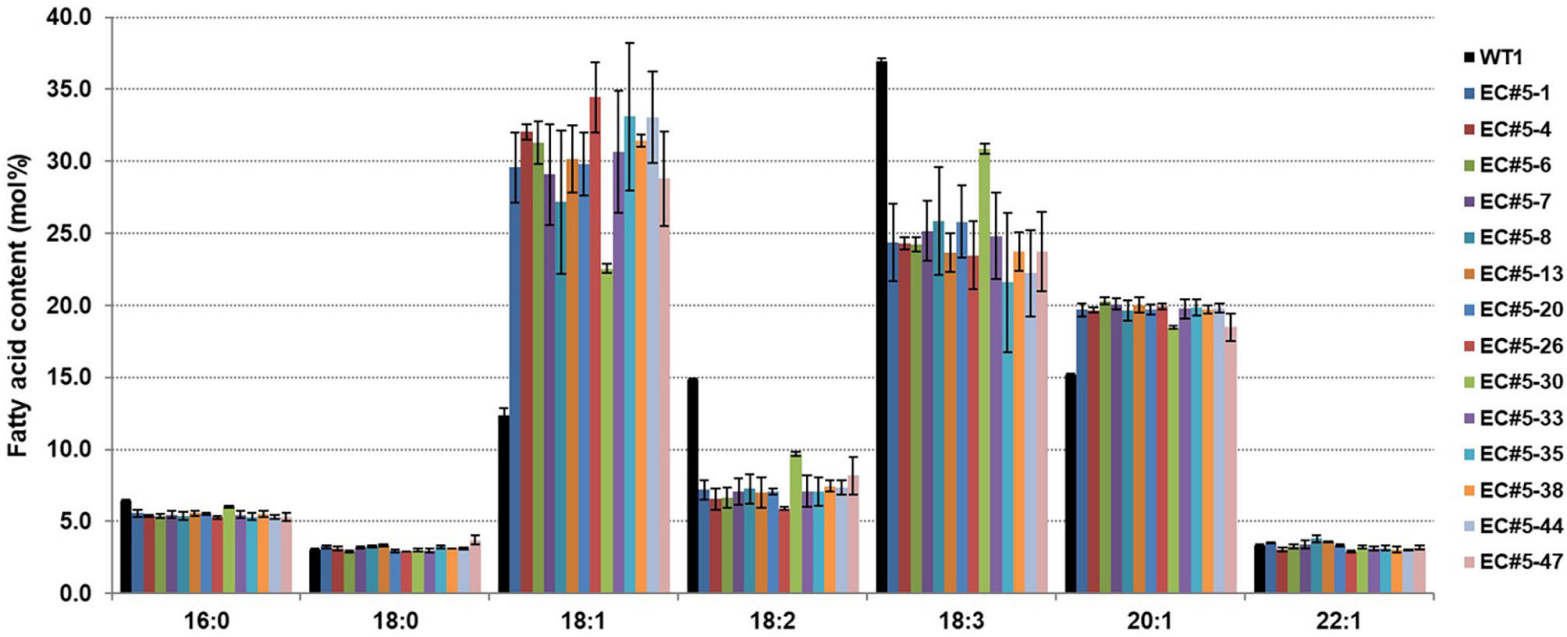
*CsFAD2* KO leads to substantial changes in fatty acid composition of Camelina seeds. Total PUFA contents are reduced and total MUFA contents are increased in *CsFAD2* KO Camelina compared to the WT. Fatty acids whose contents were <3% are not shown. Fatty acid analysis was performed in triplicate with different seeds, and error bars indicate the standard deviation.

To observe the changes in fatty acid composition in *CsFAD2* KO Camelina seeds, we performed fatty acid analysis using a gas chromatograph (GC). The total MUFA (18:1+20:1+22:1+24:1) contents were higher and total PUFA (18:2+18:3+20:2+20:3+22:3) contents were lower in the *CsFAD2* mutant Camelina compared to WT (Figure 3). The total MUFA content increased up to 57.8% and the total PUFA content decreased to a minimum of 31.7%. The PUFA content decreased by 24.6% and the MUFA content increased by 25.4% in *CsFAD2* mutant Camelina, indicating that the decrease in PUFA content indirectly led to an increase in MUFA content. However, although genotype and fatty acid composition appeared to be somewhat correlated, this could not be ascertained because the genotype may be partially different in seeds. For example, active CsFAD2 could be inactivated by Cas9 in genetically unfixed individuals. Therefore, we tried to obtain a gene-fixed individual and analyze its fatty acid composition to determine the correlation between fatty acid composition and genotype.

### The *CsFAD2* Triple Mutant Shows a Stunted Bushy Phenotype and Very Low Seed Yield

**Figure 4:**
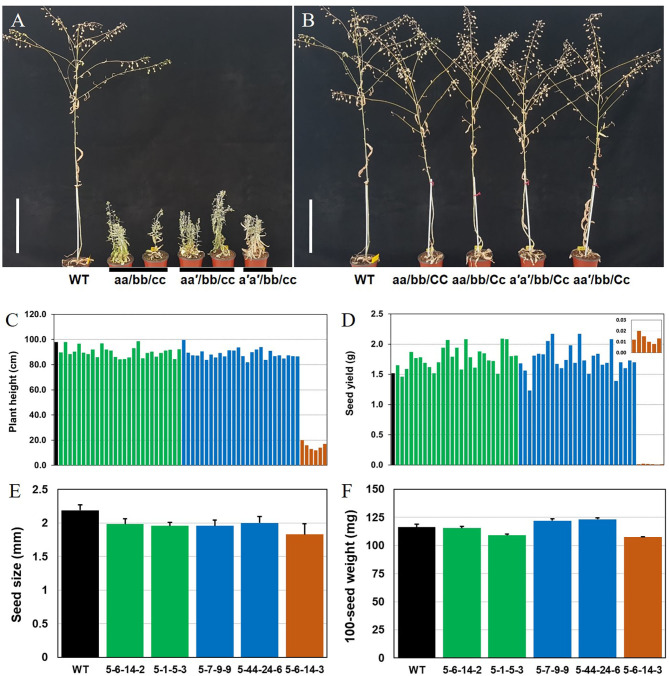
Different Camelina *CsFAD2* KO genotypes result in varying phenotypes at 3 months. **(A)** Three types of Camelina plants with complete *CsFAD2* KO showing a stunted bushy phenotype. **(B)** Camelina plants with incomplete *CsFAD2* KO and more than one normal *CsFAD2* homoeolog show normal growth. **(A–C)** indicate the *CsFAD2-1* to *CsFAD2-3* homoeologs, respectively. Lowercase letters with and without an apostrophe (′) indicate missense and non-sense mutants of *CsFAD2* homoeologs, respectively. Vertical bar = 20 cm. **(C)** Plant height (from the ground) of *CsFAD2* KO Camelina. **(D)** Seed yield (total seed weight) of *CsFAD2* KO Camelina. The line numbers of green, blue and brown bar in both **(C,D)** are in order as follows. Green bar, EC#5-44-24-3 (EC# is omitted hereafter), 5-7-9-5, 5-6-24-5, 5-6-14-6, 5-6-24-2, 5-26-10-6, 5-6-24-9, 5-26-10-7, 5-6-24-8, 5-6-24-3, 5-26-10-4, 5-7-9-7, 5-44-24-5, 5-44-24-4, 5-44-24-10, 5-7-9-6, 5-26-10-3, 5-6-24-4, 5-6-24-10, 5-1-5-8, 5-1-5-2, 5-7-9-4, 5-1-5-3, 5-6-14-2, 5-6-14-8, 5-44-24-1, and 5-26-10-10; blue bar, 5-6-14-7, 5-26-10-5, 5-6-24-6, 5-26-10-8, 5-6-14-5, 5-1-5-6, 5-7-9-10, 5-7-9-9, 5-6-24-1, 5-44-24-7, 5-6-14-10, 5-6-14-9, 5-44-24-6, 5-7-9-8, 5-7-9-1, 5-26-10-2, 5-7-9-3, 5-1-5-4, 5-44-24-9, and 5-26-10-9; brown bar, 5-1-5-5, 5-1-5-9, 5-1-5-1, 5-1-5-7, 5-6-24-7, and 5-44-24-8. **(E)** Seed size of *CsFAD2* KO Camelina (*n* = 10, Mean ± SD). **(F)** 100-seed weight of *CsFAD2* KO Camelina (*n* = 3, Mean ± SD). From **(C)** to **(F)**, black, green, blue, and brown bars represent WT, aa/bb/CC, aa/bb/Cc, and aa/bb/cc, respectively.

In the T_3_ Camelina plants, both *CsFAD2-1* and *CsFAD2-2* homoeologs were completely mutated, and *CsFAD2-3* was present as a mixture of WT, heteroallelic mutants, and homo mutants (Table 2). Regardless of whether the mutation type of *CsFAD2-1* was a homoallelic mutant or a biallelic mutant with +T and/or –CTC, all *CsFAD2* triple mutants showed a stunted bushy phenotype (Figure 4A). The plants were abnormally small and bloomed late, taking nearly a month longer than WT, with a lifespan of 100–110 days. This is similar to the finding that a dwarf *Arabidopsis* T-DNA mutant showed reduced apical dominance, decreased seed set, and slow growth (Feldmann et al., 1989). In contrast, plants with one active *CsFAD2* showed normal growth (Figure 4B). This is consistent with the earlier report that *CsFAD2* triple mutant Camelina showed inhibited growth (Morineau et al., 2017).

**Table 2:** Genotypes of T_3_ Camelina progenies revealed by Sanger sequencing following homoeolog-specific PCR.

**Line**	**FAD2-1**		**FAD2-2**		**FAD2-3**		**Genotype**
	**Zygosity**	**Mutation**	**Zygosity**	**Mutation**	**Zygosity**	**Mutation**	
EC#5-1-1	hom.	1i+1i	hom.	1i+1i	WT	WT	aabbCC
EC#5-1-4	hom.	1i+1i	hom.	1i+1i	het.	1i	aabbCc
EC#5-1-5	hom.	1i+1i	bi.	1i+5d	het.	1i	aabbCc
EC#5-1-6	hom.	1i+1i	hom.	1i+1i	WT	WT	aabbCC
EC#5-1-22	hom.	1i+1i	hom.	1i+1i	het.	1i	aabbCc
EC#5-1-24	hom.	1i+1i	bi.	1i+5d	het.	1i	aabbCc
EC#5-6-14	bi.	1i+3d	hom.	5d+5d	het.	1i	aa′bbCc
EC#5-6-18	bi.	1i+3d	hom.	5d+5d	hom.	1i+1i	aa′bbcc
EC#5-6-23	hom.	1i+1i	hom.	1i+1i	hom.	1i+1i	aabbcc
EC#5-6-24	bi.	1i+3d	hom.	5d+5d	het.	1i	aa′bbCc
EC#5-7-9	bi.	1i+3d	hom.	1i+1i	het.	1i	aa′bbCc
EC#5-7-19	hom.	1i+1i	hom.	1i+1i	hom.	1i+1i	aabbcc
EC#5-7-20	hom.	3d+3d	hom.	1i+1i	hom.	1i+1i	aabbcc
EC#5-7-21	bi.	1i+3d	hom.	1i+1i	hom.	1i+1i	aa′bbcc
EC#5-12-5	hom.	3d+3d	hom.	1i+1i	hom.	1i+1i	a′a′bbcc
EC#5-20-8	hom.	1i+1i	bi.	1i+5d	WT	WT	aabbCC
EC#5-26-3	bi.	1i+3d	hom.	1i+1i	WT	WT	aa′bbCC
EC#5-26-10	bi.	1i+3d	hom.	1i+1i	het.	1i	aa′bbCc
EC#5-26-20	bi.	1i+3d	hom.	1i+1i	het.	1i	aa′bbCc
EC#5-26-22	hom.	1i+1i	hom.	1i+1i	hom.	1i+1i	aabbcc
EC#5-26-24	bi.	1i+3d	hom.	1i+1i	hom.	1i+1i	aa′bbcc
EC#5-35-4	bi.	1i+3d	hom.	5d+5d	het.	1i	aa′bbCc
EC#5-35-15	hom.	1i+1i	hom.	5d+5d	hom.	1i+1i	aabbcc
EC#5-35-17	hom.	3d+3d	hom.	5d+5d	het.	1i	a′a′bbCc
EC#5-35-24	bi.	1i+3d	hom.	5d+5d	hom.	1i+1i	aa′bbcc
EC#5-44-3	hom.	3d+3d	bi.	1i+5d	het.	1i	a′a′bbCc
EC#5-44-11	hom.	3d+3d	bi.	1i+5d	hom.	1i+1i	a′a′bbcc
EC#5-44-15	hom.	3d+3d	hom.	5d+5d	hom.	1i+1i	a′a′bbcc
EC#5-44-20	hom.	3d+3d	hom.	1i+1i	hom.	1i+1i	a′a′bbcc
EC#5-44-24	hom.	3d+3d	bi.	1i+5d	het.	1i	a′a′bbCc

*1i, 3d, and 5d indicate 1 nt insertion, 3 nt deletion, and 5 nt deletion, respectively. Bi., het., and hom. represent biallelic, heteroallelic, and homoallelic mutants, respectively. Uppercase letters, lowercase letters, and lowercase letters with ′ in the Genotype column represent an intact CsFAD2 homoeolog, a nonsense CsFAD2 mutant gene, and a missense CsFAD2 mutant gene with a 3 nt deletion, respectively. A, B, and C in the Genotype column represent CsFAD2-1, CsFAD2-2, and CsFAD2-3, respectively*.

We measured the correlations between plant height and seed yield or fatty acid content vs. genotype in T_4_ plants which were performed genotyping (Supplementary Table 5). Fatty acid analysis clearly distinguished the plants based on the number of active *CsFAD2* homoeologs, whereas phenotypes such as plant height and seed yield did not show a correlation with genotype (Figures 4C,D). *CsFAD2* KO Camelina plants appeared to be slightly shorter than WT, but the difference was not significant. The plant height of *CsFAD2* triple mutants was also extremely reduced, reaching at most 20% that of the WT (Figure 4C). Meanwhile, the seed yield of the *CsFAD2* triple mutants decreased to 1% that of the WT (Figure 4D).

Seed size of *CsFAD2* KO Camelina was slightly smaller than that of WT Camelina but the difference was not significant (Figure 4E). In the case of *CsFAD2* triple mutant EC#5-6-14-3, its seed size was 86% level compared to that of WT, which is not a dramatic reduction but showing the difference between their seed sizes. One-hundred-seed weight of *CsFAD2* KO Camelina was also determined (Figure 4F). Compared to 100-seed weight of WT, that of EC#5-6-14-2 was almost same, that of EC#5-7-9-9 and EC#5-44-24-6 was slightly higher, and that of RC5-1-5-3 and EC#5-6-14-3 was slightly lower. It did not show the correlation between seed weight and genotype of *CsFAD2* gene.

Several lines of argument indicated that this phenotype was not due to an off-target effect. First, this phenotype only appeared when all three *CsFAD2* homoeologs were KO. Second, the same phenotype was seen in triple mutant Camelina of *CsFAD2* homoeologs generated using different sgRNAs in a previous study (Morineau et al., 2017).

### Seeds and Leaves of *CsFAD2* Triple Mutant Camelina Show Significantly Increased MUFA and Decreased PUFA Contents

**Figure 5:**
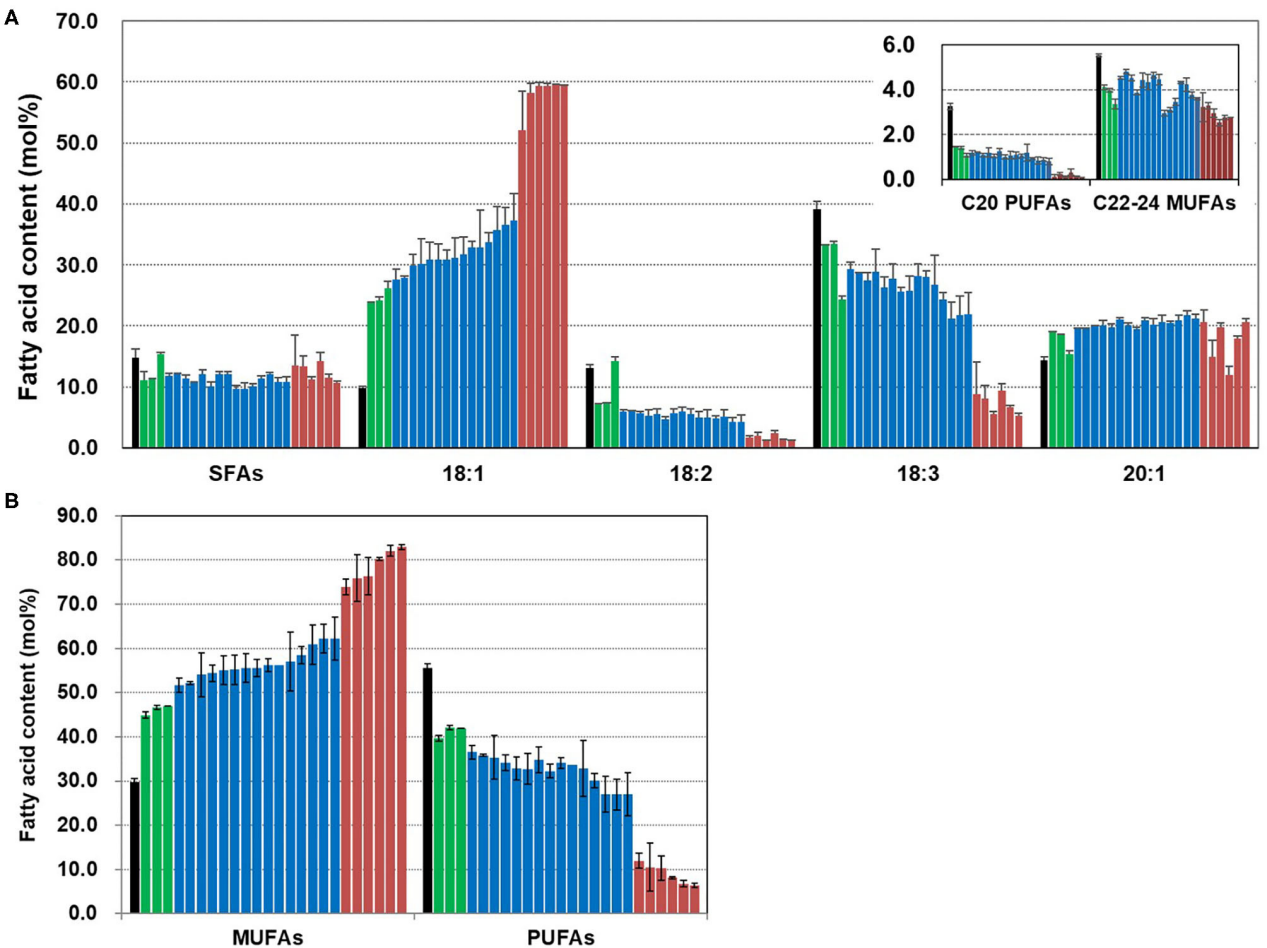
Fatty acid analysis of *CsFAD2* KO T_4_ Camelina seeds. **(A)** Fatty acid composition of *CsFAD2* KO Camelina seeds showing high-oleic and low-PUFA traits. SFAs, saturated fatty acids containing 16:0, 18:0, 20:0, 22:0, and 24:0; C20 PUFAs, 20:2, and 20:3; C22-24 MUFAs, 22:1, and 24:1. **(B)** MUFA and PUFA contents of *CsFAD2* KO Camelina seeds. Black, green, blue, and brown bars represent WT, aa/bb/CC, aa/bb/Cc, and aa/bb/cc, respectively. The line numbers of green, blue and brown bars are in order as follows. Green bar, EC#5-1-5-2 (EC# is omitted hereafter), 5-6-14-2, and 5-6-24-4; blue bar, 5-1-6-1, 5-6-14-7, 5-6-2-41, 5-7-9-3, 5-26-10-5, 5-26-10-6, 5-44-24-4, 5-44-24-6, 5-6-24-6, 5-26-10-8, 5-6-14-5, 5-1-5-6, 5-7-9-10, 5-7-9-9, and 5-6-24-1; brown bar, 5-1-5-5, 5-1-5-9, 5-1-5-1, 5-1-5-7, 5-6-24-7, and 5-44-24-8. Fatty acid composition was analyzed in triplicate, and error bars indicate standard deviation.

We analyzed the fatty acid composition of seeds from T_4_ plants and the contents of MUFAs and PUFAs (Figure 5). Overall saturated fatty acid content was slightly reduced in the *CsFAD2* triple mutants compared to the WT. The difference in 18:1 content was greater than the difference in the contents of other fatty acids. The 18:1 content increased 6.1-fold to a maximum of 59.5% in the mutants compared to 9.8% in the WT. This was expected to some extent because the decrease in 18:2 and 18:3 content due to *CsFAD2* KO led directly to an increase in 18:1 content. However, the 20:1 content increased only 6.3%, and the content of C22-24 MUFAs decreased slightly. C20 PUFA levels were low in both mutant and WT seeds, but decreased by more than half in the mutant. All of these change of fatty acid composition is consistent with recent findings for *CsFAD2* KO Camelina (Morineau et al., 2017).

We sorted the *CsFAD2* KO mutants according to MUFA content from lowest to highest. The MUFA content was divided discontinuously according to the degree of mutation of the *CsFAD2* homoeologs (Figure 5B). All plants besides WT were KO mutants of both *CsFAD2-1* and *CsFAD2-2* homoeologs. The MUFA content of the *CsFAD2-1/CsFAD2-2* double KO mutant was 44.9–47%; those of the *CsFAD2-1*/*CsFAD2-2* double KO and *CsFAD2-3* heteroallelic mutants ranged from 52.2 to 62.2%; and that of the *CsFAD2* triple mutant was 73.8–82.9%. We investigated whether the wide range of different MUFA contents was due to the difference in mutation type between a non-sense and missense mutation. Among *CsFAD2-1*/*CsFAD2-2* double KO and *CsFAD2-3* heteroallelic mutants, the MUFA content was 54.0–60.9% in the +A homoallelic mutant of *CsFAD2-1*, 52.2–62.2% in the biallelic mutant, and 55.5–56.2% in the –CTC homoallelic mutant. In addition, among *CsFAD2* triple mutants, MUFA content was 75.9–82.9% in the +A homoallelic mutant of *CsFAD2-1*, 77.2–82.1% in the biallelic mutant, and 73.8–80.3% in the –CTC homoallelic mutant. There were differences in MUFA content within the same mutation type, but these showed little consistency. Therefore, although CsFAD2 activity has a significant influence on fatty acid composition, it appears that the type of mutation in *CsFAD2* homoeologs does not affect fatty acid composition.

**Figure 6:**
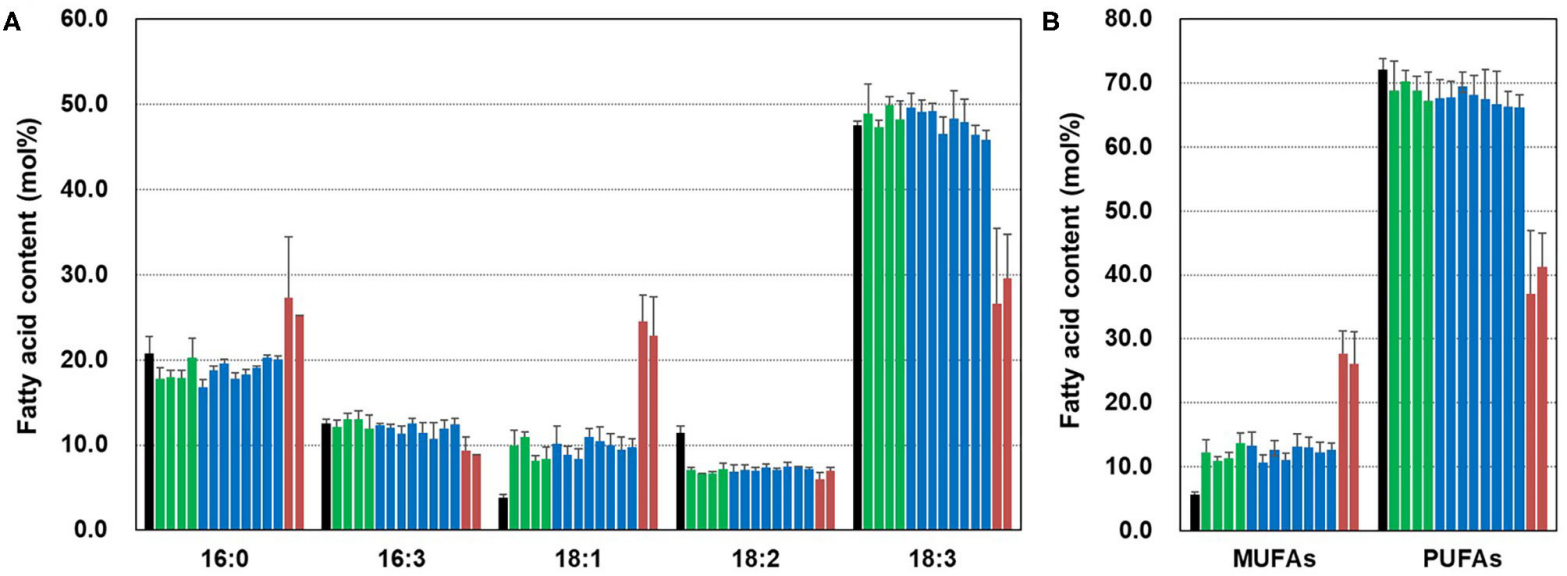
Fatty acid analysis of *CsFAD2* KO T_4_ Camelina leaves. **(A)** Fatty acid composition of *CsFAD2* KO Camelina leaves. **(B)** MUFA and PUFA contents of *CsFAD2* KO Camelina leaves. Minor peaks, 16:1, 16:2, and 18:0 peaks were omitted. Black, green, blue, and brown bars represent WT, aa/bb/CC, aa/bb/Cc, and aa/bb/cc, respectively. The line numbers of green, blue and brown bars are in order as follows. Green bar, EC#5-1-5-2 (EC# is omitted hereafter), 5-6-14-2, 5-6-24-4, and 5-7-9-4; blue bar, 5-1-6-1, 5-6-14-7, 5-6-2-41, 5-7-9-3, 5-26-10-5, 5-26-10-6, 5-44-24-4, and 5-44-24-6; brown bar, 5-26-10-1, and 5-6-14-3. Fatty acid composition was analyzed in triplicate, and error bars indicate standard deviation.

To examine the effect of *CsFAD2* KO on the fatty acid composition of Camelina leaves, we extracted fatty acids from the leaves and analyzed the fatty acid composition. For simplicity, the WT *CsFAD2-1, CsFAD2-2*, and *CsFAD2-3* homoeologs are represented hereafter as A, B, and C, respectively, and mutated versions with lower-case letters (Table 2). Compared to WT, aa/bb/CC and aa/bb/Cc plants showed at least a 2-fold increase in 18:1 content and an ~5% reduction in 18:2 content, but there was no difference among individuals with different numbers of functional *CsFAD2-3* alleles (i.e., CC vs. Cc). Compared to WT, aa/bb/CC and aa/bb/Cc plants showed little or no difference in 16:0, 16:3, or 18:3 content. However, *CsFAD2* triple mutants showed dramatic changes in 18:1 and 18:3 contents, as well as changes in 16:0 and 16:3 contents (Figure 6A). The 18:1 content of the *CsFAD2* triple mutants increased 6.5-fold, i.e., 24.5% compared to 3.8% in the WT, whereas the 18:3 content decreased to almost half (26% from 47.5% in the WT). The 16:0 content of *CsFAD2* triple mutants increased 1.3-fold (27.3% from 20.8% in the WT), whereas the 16:3 content decreased (9.3 vs. 12.5% in the WT). We also examined MUFA and PUFA contents in the triple mutants. The PUFA contents of both aa/bb/CC and aa/bb/Cc decreased by up to 6% compared to the WT. On the other hand, the PUFA content of the *CsFAD2* triple mutants decreased to 37–41% (from 72% in the WT; Figure 6B). These results suggest that the difference in PUFA contents, especially 18:3 content, between WT and *CsFAD2* mutant leaves was strongly correlated with the difference in growth between WT and *CsFAD2* mutants (Figure 4).

The fatty acid composition of seeds was dependent on the number of active *CsFAD2* homoeologs, whereas membrane lipid composition in leaves was dependent on the presence or absence of CsFAD2 activity. A dramatic change in 18:3 fatty acid content in chloroplasts affects agronomic traits such as plant height and seed yield (Figures 4C,D, 6). Therefore, our findings suggest that the critical decrease in the 18:3 content of phospholipids from the endoplasmic reticulum to chloroplast in response to the complete KO of *CsFAD2* causes a decrease in the 18:3 content of chloroplast lipids, thereby leading to growth inhibition and extremely low seed yields.

When the only *FAD2* gene in *Arabidopsis* was knocked out, the stem length was slightly reduced at 22°C, and the plants barely grew at 12°C and did not survive at 6°C (Miquel et al., 1993). However, it seemed unusual that *CsFAD2* triple mutant Camelina exhibited a stunted bushy phenotype.

*fad2*/*fae1* double mutant, which shows a stunted bushy phenotype and reduced seed production (James and Dooner, 1991). Similarly, *fab2 Arabidopsis* shows a miniature growth phenotype due to increased stearic acid (18:0) levels (Lightner et al., 1994a,b).

To explore why *CsFAD2* triple mutant Camelina had a different phenotype from *fad2 Arabidopsis*, we investigated the fatty acid composition of leaf lipids from WT and mutant *Arabidopsis* and Camelina and calculated the degree of unsaturation of total fatty acids (UDTFA) as follows: UDTFA = [total saturated fatty acids (%)^*^0] + [total monenoic acids (%)^*^1] + [total dienoic acids (%)^*^2] + [total trienoic acids (%)^*^3]. The fatty acid composition and UDTFA values are listed in Supplementary Table 5. The UDTFA values of WT *Arabidopsis* and Camelina were 219.2 and 209.8, respectively, whereas those of *fad2* mutant *Arabidopsis* and Camelina were 205.5 and 158.6, respectively. In the *Arabidopsis fad2* mutant, the UDTFA value was 94% of the WT level, indicating that the PUFA content was not significantly reduced. The leaf lipids of *fad2* and WT *Arabidopsis* contained 40.5% and 46.0% 18:3, respectively (Okuley et al., 1994). By contrast, the Camelina *CsFAD2* triple mutant had a UDTFA value 76% of the WT level. Leaf lipids of the *CsFAD2* triple mutant contained up to 29.6% 18:3, whereas leaf lipids of WT Camelina contained 47.5% 18:3 (Figure 6A). In *Arabidopsis fab2*, due to the increased 18:0 level (Lightner et al., 1994b), UDTFA was reduced by 86% compared to WT *Arabidopsis*. The exact level needed to inhibit plant growth is unknown. However, the degree of unsaturation in *CsFAD2* KO Camelina was significantly lower than that in *fad2 Arabidopsis*, which is likely to be a major factor in the stunted phenotype.

18:3 fatty acids in chloroplasts are generated via two pathways. First, 18:1-galactolipids are synthesized in the chloroplast and unsaturated to form 18:3 by a sequential reaction catalyzed by FAD6 and FAD7/8 (Browse et al., 1989; Iba et al., 1993; Yadav et al., 1993; Gibson et al., 1994). Second, 18:3-phospholipids synthesized in the endoplasmic reticulum are transported to the chloroplast and sequentially converted into DAG and galactolipids (Browse and Somerville, 1991). Even though the chloroplast desaturases FAD6 and FAD7/8 synthesize 18:2 and 18:3 PUFAs incorporated in galactolipids, respectively, the complete deficiency of CsFAD2 activity caused a decrease in 18:3 and increase in 16:0 fatty acids in chloroplasts in leaves (Figures 4A, 6A).

Given that the fatty acid compositions of WT *Arabidopsis* and Camelina leaves are normally quite similar, it was unclear why they differed strongly when *FAD2* was mutated. We considered two explanations. First, FAD6 and FAD7/8 activity might be much higher in *Arabidopsis* than in Camelina. Therefore, in Camelina, even if fewer monounsaturated fatty acids were incorporated into phospholipids and transported to chloroplasts due to *CsFAD2* KO, it might be impossible to raise the 18:3 content in chloroplast lipids to WT levels. Second, although the activities of FAD6 and FAD7/8 in *Arabidopsis* and Camelina may be similar, these genes may be upregulated in *fad2 Arabidopsis* to compensate for the reduced 18:3 content but downregulated in *CsFAD2* KO Camelina. This could be inferred based on the changes in leaf fatty acid composition. In *fad2 Arabidopsis*, 16:0 levels decreased and 16:3 levels increased compared to WT, which appeared to increase the desaturase activity in chloroplasts. In *CsFAD2* KO Camelina, 16:0 levels increased and 16:3 levels decreased compared to WT, resulting in decreased desaturase activity in the chloroplast. However, finding the exact reason for these differences will require experimental validation.

Kang et al. (2011) examined the tissue-specific expression patterns of the *CsFAD2* homoeologs in Camelina. *CsFAD2-1* expression was constitutive in all tissues and was the highest for three homoeologs. *CsFAD2-2* expression was generally weak, especially in leaf and root tissues. However, *CsFAD2-2* expression was the strongest in developing seeds, and *CsFAD2-2* was considered to be a seed-specific *FAD2*. *CsFAD2-3* had similar expression patterns to *CsFAD2-2*, such as weak expression in leaf and root tissues, but the expression intensity was generally stronger than that of *CsFAD2-2*. In addition, an analysis of MUFA contents in three combinations of *CsFAD2* double mutants in which only one *CsFAD2* homoeolog was active, that is, AA/bb/cc, aa/BB/cc, and aa/bb/CC, revealed that the MUFA contents of AA/bb/cc and aa/BB/cc leaves were similar, but the MUFA contents of aa/bb/CC leaves were slightly higher than for the others, whereas the MUFA contents in seeds were highest in aa/BB/cc, followed by AA/bb/cc and aa/bb/CC (Morineau et al., 2017). These results suggest that the expression levels of *CsFAD2* homoeologs in seeds decrease from aa/BB/cc to AA/bb/cc to aa/bb/CC, that is, from active homoeolog *CsFAD2-2* to *CsFAD2-1* to *CsFAD2-3*.

### Indels of *FAD2* Homoeologs Lead to a Truncated Protein or Single Amino Acid Deletion

**Figure 7:**
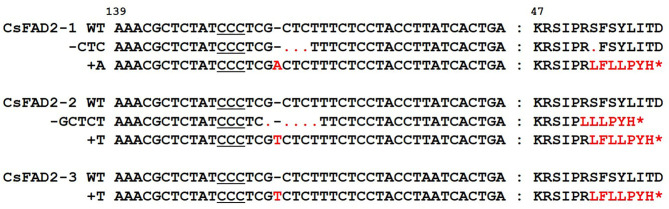
Nucleotide sequences and deduced amino acid sequences of mutants from three *CsFAD2* homoeologs. Red font indicates different nucleotides or amino acids compared to WT. Dots and asterisks represent gaps produced by deletion and the termination of translation, respectively. The PAM sequence used in this study is underlined.

To examine how the mutations of the three *CsFAD2* homoeologs affected its amino acid sequence, we predicted the translated amino acid sequences based on the nucleotide sequences obtained by Sanger sequencing and examined by multiple sequence alignment (Figure 7). In mutation 3d in *CsFAD2-1* (see Table 1 for definitions of mutations), deletion of CTC at the 3′ position of the DSB resulted in the deletion of S^53^ from the amino acid sequence without a frameshift and early termination of translation. 1i of *CsFAD2-1* caused a frameshift by inserting A at the DSB, resulting in the formation of an aberrant protein with premature termination of translation. A frameshift also occurred between 5d and 1i of *CsFAD2-2* and in 1i of *CsFAD2-3*, resulting in truncated proteins. Both 1i of *CsFAD2-2* and *CsFAD2-3* differed from 1i of *CsFAD2-1*, with T and A insertions. Indels in which the number of nucleotides is not a multiple of three generally cause a frameshift, resulting in a truncated protein. However, indels in which the number of nucleotides affected is a multiple of three result in the deletion of whole triplet codons, so that only some amino acids are deleted. Therefore, gene KO is not expected in this case. However, in the current study, CsFAD2 activity was abolished in the presence of the –CTC mutation, as for non-sense mutations such as +A, +T, and –GCTCT. Morineau et al. (2017) reported that six non-frameshift mutants occurred between amino acid codons 187 to 201, involving the fourth transmembrane domain of *CsFAD2* homoeologs. It is possible that the non-frameshift mutants lost their original three-dimensional structure and activity due to the collapse of the fourth transmembrane domain. To determine whether the deleted amino acid, S^53^, is a crucial part of the CsFAD2 structure, we performed a search with the transmembrane domain prediction tool by querying with WT *CsFAD2-1* and the *CsFAD2-1* mutant with –CTC, respectively. In WT, S^53^ is located in front of the first transmembrane domain starting from the 54th or 55th amino acid, and in the –CTC variant, the first transmembrane domain starts from the 53rd or 54th amino acid. Therefore, this mutation does not seem to cause a major structural difference in the protein. In addition, S^53^ is outside the His boxes of the three FAD2 proteins, at positions 106-111, which have the sequence HECGHH and are essential for the protein's desaturase function (Shanklin et al., 1994; Kurdrid et al., 2005). Therefore, the deletion of S^53^ does not appear to directly affect FAD2 function. It is likely that this deletion affects FAD2 activity by some other mechanism, but the nature of this effect remains to be revealed.

## Conclusion

By generating *CsFAD2* KO using CRISPR-Cas9, we increased the MUFA content of Camelina seeds, but this was accompanied by growth inhibition and deterioration of other agronomic traits. It is difficult to increase the MUFA level above 80% and maintain a normal phenotype. However, if the gene(s) involved in PUFA accumulation are also mutated to the *CsFAD2* double mutant form (aa/bb/CC), and/or if the gene(s) involved in PUFA biosynthesis in chloroplasts are overexpressed in the *CsFAD2* triple mutant form (aa/bb/cc), it should be possible to generate Camelina with enhanced MUFA content and normal growth. This would help expand the use of Camelina seed oil, which is already widely used for industrial purposes such as biofuels. Perhaps this method could also be used for the production of unusual fatty acids, such as hydroxy fatty acids, that are generated from MUFAs as precursors.

## Data Availability Statement

The raw data supporting the conclusions of this article will be made available by the authors, without undue reservation.

## Author Contributions

K-RL, IJ, and S-GK performed vector construction. K-RL, IJ, HY, and JL performed fatty acid analysis, genomic DNA extraction, genomic PCR, and analysis of Sanger sequencing results. S-GK selected the sgRNA sequence. K-RL, IJ, and S-GK conducted targeted deep sequencing. IJ, H-SK, S-JA, and S-KL performed Camelina transformation and selected transgenic plants/seeds. K-RL and HUK analyzed the data. K-RL wrote the paper. All authors read and approved the final manuscript.

## Conflict of Interest

The authors declare that the research was conducted in the absence of any commercial or financial relationships that could be construed as a potential conflict of interest.
